# Antileukemic Efficacy of Monomeric Manganese-Based Metal Complex on KG-1A and K562 Cell Lines

**DOI:** 10.1155/2013/709269

**Published:** 2013-10-08

**Authors:** Sandeep Kumar Dash, Sourav Chattopadhyay, Totan Ghosh, Satyajit Tripathy, Sabyasachi Das, Debasis Das, Somenath Roy

**Affiliations:** ^1^Immunology and Microbiology Laboratory, Department of Human Physiology with Community Health, Vidyasagar University, Midnapore, West Bengal 721 102, India; ^2^Department of Chemistry, University of Calcutta, 92 A. P. C. Road, Kolkata 700 009, India

## Abstract

Transitional metals and metal compounds have been used in versatile platforms for biomedical applications and therapeutic intervention. Severe side effects of anticancer drugs produce an urgent urge to develop new classes of anticancer agents with great potency as well as selectivity. In this background, recent studies demonstrate that monomeric manganese (MnII) thiocyanate complex (MMTC) holds great promise to exert effective antileukemic effects. MMTC was developed by a simple chemical reaction and characterized by elemental analyses, thermal analyses, and Fourier transform infrared (FTIR) spectroscopy. Anti-leukemic efficacy of the developed MMTC was estimated in KG-1A (AML) and K562 (CML) cell lines. Cell viability study, drug uptake assay, cellular redox balance (GSH and GSSG level), nitric oxide (NO) release level, reactive oxygen species (ROS) formation, alteration of mitochondrial membrane potential (MMP), and DNA fragmentation revealed that MMTC was able to produce significant antiproliferative effects on both cell lines at 25 **μ**g mL^−1^ without showing any toxicological impact on normal lymphocytes. These findings will enlighten the biomedical application of manganese-based metal complexes as anti-leukemic agents.

## 1. Introduction

Leukemia is a type of cancer of the blood or bone marrow, characterized by an abnormal increase of immature white blood cells called “blasts” [1]. Acute myelogenous leukemia (AML) is a fast growing fatal form of leukemia which produces immature white blood cells, begins in bone marrow cells, and spreads into the blood system. Chronic myelogenous leukemia (CML) is an uncommon type of leukemia, making up about 15% of all the cases of leukemia among adults, results from a somatic mutation in a pluripotential lymphohematopoietic cell, and thereby produces large number of white blood cells [2]. The modern research regarding the development of the metal-based anticancer drugs began with the discovery of the platinum (II) complex cisplatin by Rosenberg in the 1960s [3]. Metal complex or coordination compound is a structure consisting of a central metal atom that remains surrounded by molecules or anions. Transition metal complexes have an esteemed role in antitumor therapy and open a new area of research in the field of medicinal chemistry [4]. Nowadays, metal ion complexes had quickly turned out to be an interesting and attractive compounds in the development of anticancer drugs due to their unique chemical reactivity [5]. This phenomenon has started the development of metal-based drugs with promising pharmacological application and may offer unique therapeutic opportunities [6]. The use of metal complexes as therapeutic agents has become more pronounced due to their diverse mode of action not only in cancer therapy but also in that they can be used as anti-inflammatory, anti-infective, and antidiabetic compounds [7]. Many cytotoxic metal complexes target DNA as they exert a great role in replication and cell viability. Another important characteristic of metal complexes is their potentiality to alter the cellular redox homeostasis by switching between several oxidation states [8]. Cancer cells are different from healthy cells due to their unique redox balances and the generation of high level of reactive oxygen species (ROS). Consequently, metal complexes interfere with the cellular redox homeostasis, stimulate generation of ROS, and interact with DNA strands and thereby produce antiproliferative activity against cancer cells. In a biological system, manganese (transitional metal) plays a great role as an ever-increasing number of biomolecules were found to contain manganese, including manganese-containing superoxide dismutase (Mn-SOD), catalase, Mn-ribonucleotide reductase, and Mn-peroxidase. Mn-SOD actively eliminates harmful free radicals from cellular systems [9]. So, Mn containing complexes may be a valuable agent in antileukemic therapy. To the best of our knowledge, still now no work has been carried out regarding the anti-leukemic potential of monomeric manganese thiocyanate complex (MMTC). Considering this background, the present work has been carried out to develop the MMTC and to evaluate its anti-leukemic activity in human acute myelogenous leukemia (AML) and human chronic myelogenous leukemia (CML) cell lines. Doxorubicin (DOX) has been included in this study as a positive control drug.

## 2. Materials and Methods

### 2.1. Culture Media and Chemicals

Manganese (II) perchlorate hexahydrate and 2-benzoylpyridine were purchased from Aldrich Chemical Company and used as received. Crystal violet solution, Histopaque 1077, and Rhodamine B were procured from Sigma. RPMI 1640, penicillin, streptomycin, and doxorubicin were procured from Sigma (St. Louis, MO, USA). Fetal bovine serum (FBS) was purchased from Gibco/Invitrogen. Sodium chloride (Nacl), sodium carbonate (Na_2_CO_3_), sucrose, Hanks balanced salt solution (HBSS), HEPES-Na+ buffer, and 3-[4,5-dimethylthiazol-2-yl]-2,5-diphenyltetrazolium bromide (MTT) were purchased from Himedia, India. Tris-HCl, Tris buffer, KH_2_PO_4_, K_2_HPO_4_, HCl, formaldehyde, alcohol, Triton X-100, sodium dodecyl sulphate (SDS), phenol, chloroform, isoamyl alcohol, ethidium bromide (EtBr), 2-vinylpyridine and other chemicals were procured from Merck Ltd., SRL Pvt. Ltd., Mumbai, India. Commercially available dimethyl sulfoxide (DMSO) was procured from Hi-media, India, and was purified by vacuum distillation over KOH. All other chemicals were from Merck Ltd., SRL Pvt. Ltd., Mumbai, India, and were of the highest purity grade available. Solvents were dried according to the standard procedure and distilled prior to use [10].

### 2.2. Preparation of Mononuclear Complex [Mn(Phpyk)_2_(SCN)_2_]

Mononuclear complex [Mn(Phpyk)_2_(SCN)_2_] (MMTC) was prepared according to our previously reported method [11]. In brief, a methanolic solution (5 mL) of 2-benzoylpyridine (Phpyk) (0.366 g; 2 mmol) was added dropwise with constant stirring to 10 mL methanolic solution of manganese (II) perchlorate hexahydrate (1 mmol; 0.362 g). The stirring was continued for further 1 h, and then, an aqueous solution (5 mL) of sodium thiocyanate (0.162 g; 2 mmol) was added dropwise. After 2 h stirring, the resulting mixture was filtered and the filtrate was kept in a CaCl_2_ desiccator.

### 2.3. Physical Measurements

Elemental analyses (carbon, hydrogen, and nitrogen) were performed using a Perkin-Elmer 240C elemental analyzer. Thermal analyses (TGA) were carried out on a TGA/SDTA851e Mettler-Toledo thermal analyzer. For the TGA test, the sample was heated from room temperature to 700°C under flowing nitrogen atmosphere (flow rate: 40 cm^3 ^min^−1^) at a heating rate of 10°C min^−1^ in a platinum crucible. To determine the structural features of the samples, Fourier transform infrared (FTIR) spectroscopy was carried out at 25°C using a Perkin-Elmer Spectrum RXIFT-IR System FT-IR spectrometer with 64 scans for wave numbers ranging from 400 to 4000 cm^−1^ and resolution 4 cm^−1^. The KBr pellet method was used to prepare the samples [11].

### 2.4. Cell Lines Culture and Maintenance

KG-1A and K562 cell lines were obtained from NCCS, Pune, India. All cell lines were cultivated and maintained in RPMI-1640 complete media supplemented with 10% heat inactivated fetal bovine serum (FBS), 2 mM glutamine, 100 U mL^−1^ penicillin, 100 *μ*g mL^−1^ streptomycin, and 4 mM L-glutamine under 5% CO_2_ and 95% humidified atmosphere at 37°C in a CO_2_ incubator. Cells were cultured and maintained in logarithmic growth phase until the number of cells reaches 1.0 × 10^6^ cells mL^−1^.

### 2.5. Selection of Human Subjects for the Collection of Lymphocytes

Six healthy subjects were chosen to collect the blood sample for separation of lymphocytes. All subjects enrolled in this study were asymptomatic, and none of them had abnormality on physical examinations and routine laboratory tests. The subjects were from same geographical area and same economic status, nonsmokers and nonalcoholic, and having the same food habit. These subjects received no medication, including vitamin E and vitamin C. All subjects gave informed consent. The selection excluded not only individuals with acute infections or chronic diseases, but also healthy individuals undergoing supplementation with antioxidants. The study protocol was in accordance with the declaration of Helsinki, and was approved by the institutional ethical committee of Vidyasagar University [12].

### 2.6. Isolation of Peripheral Blood Lymphocytes

Blood samples were collected from these six healthy human volunteers by venipuncture in 5 mL heparin coated vacutainers satisfying the method of Hudson and Hay [13]. Five milliliters of blood was diluted (1 : 1) with phosphate buffered saline (PBS) and layered onto Histopaque 1077 (Sigma) by using a Pasteur pipette and centrifuged at 400 ×g (1500 rpm) for 40 min at room temperature. The upper monolayer of buffy coat, that is, lymphocytes, was transferred using a clean Pasteur pipette to a clean centrifuge tube and washed three times in a balanced salt solution. The lymphocytes were resuspended in RPMI complete media supplemented with 10% FBS and incubated for a day at 37°C in a 95% air/5% CO_2_ atmosphere in a CO_2_ incubator.

### 2.7. Drug Preparation

A 10 mg/mL stock of MMTC was prepared by dissolving 10 mg of MMTC in DMSO. It was then serially diluted with RPMI media to prepare working concentrations. The amount of DMSO for each concentration, never exceeded >0.75%.

### 2.8. Experimental Design

Each type of cells was divided into 9 groups. Each group contained six (6) petri dishes (2 × 10^5^ cells in each). The cells of each petri dish of control and experimental groups were maintained in RPMI 1640 media supplemented with 10% FBS, 50 *μ*g mL^−1^ gentamicin, 50 *μ*g mL^−1^ penicillin, and 50 *μ*g mL^−1^ streptomycin at 37°C in a 95% air/5% CO_2_ atmosphere in a CO_2_ incubator. The following groups were considered for the experiment and cultured for 24 h:

 Group I: control, that is, cells + culture media;
 Group II: cells + 1 *μ*g mL^−1^ doxorubicin in culture media;
 Group III: cells + 1 *μ*g mL^−1^ MMTC in culture media;
 Group IV: cells + 10 *μ*g mL^−1^ doxorubicin in culture media;
 Group V: cells + 10 *μ*g mL^−1^ MMTC in culture media;
 Group VI: cells + 25 *μ*g mL^−1^ doxorubicin in culture media;
 Group VII: cells + 25 *μ*g mL^−1^ MMTC in culture media;
 Group VIII: cells + 50 *μ*g mL^−1^ doxorubicin in culture media;
 Group IX: cells + 50 *μ*g mL^−1^ MMTC in culture media.

After the treatment schedule, the cells were collected from the petri dishes separately and centrifuged at 2,200 RPM for 10 min at 4°C to separate cells and sups [12]. The cells were washed twice with 50 mM PBS, pH 7.4. A required amount of cells was lysed using hypotonic lysis buffer (10 mM TRIS, 1 mM EDTA, and Triton X-100, pH 8.0) for 45 min at 37°C and then processed for the biochemical estimation [14]. Intact cells were used for determination of ROS, mitochondrial membrane potential, and different microscopic observations.

### 2.9. *In Vitro* Cell Proliferation Assay

The cytotoxicity of MMTC was quantitatively estimated by nonradioactive, colorimetric assay systems using tetrazolium salt, 3-[4,5-dimethylthiazol-2-yl]-2,5-diphenyltetrazolium bromide (MTT). Briefly, MTT was dissolved in phosphate-buffered saline at 5 mg mL^−1^ and filter sterilized to remove the small amount of insoluble residue present in some batches. The MTT solution was then added directly to all appropriate microtiter plate wells (10 *μ*L per 100 *μ*L medium) containing cells and complete growth medium, with or without the nanoparticle. The plate was then incubated for 4 h at 37°C to allow MTT to metabolize to formazan. Subsequently, the supernatant was aspirated, and 100 *μ*L of HCl-isopropanol solution (1 : 1) was added to each culture plate and mixed thoroughly to dissolve the dark blue formazan crystals. The optical density (OD) was measured on ELISA reader (Bio-Rad, Model 550, Tokyo, Japan) using test and reference wavelengths of 570 and 630 nm, respectively [14]. The percentage of proliferation was calculated by using the following equation:

(1) %Proliferation=[OD  sample−OD  control]OD  control×100.

### 2.10. *In Vitro* Drug Uptake Assay

*In vitro* MMTC uptake assay was performed according to standard methods [15] with some modifications. Briefly, cells were plated at a density of 2.5 × 10^4^ cells/Petridis (35 mm) containing 12 mm sterile cover slips for 24 h. Rhodamine B tagged MMTC at 10 *μ*g/mL concentration were incubated for 6 h at 37°C in a 95% air/5% CO_2_ atmosphere in a CO_2_ incubator. After a defined time, the cover slips were removed; the cells were washed 2 times with PBS and immediately observed in green light under the fluorescence microscope (Nikon Eclipse LV100POL) for uptake assessment. Images were acquired at 50x optical zoom, and analysis was done using ImageJ software v.r. 1.43 (NIH).

### 2.11. Determination of Reduced Glutathione (GSH)

Reduced glutathione estimation in cell lysate was performed by the method of Dey and Roy [16]. The required amount of the sample was mixed with 25% of TCA and centrifuged at 2,000 ×g for 15 min to settle down the precipitated proteins. The supernatant was aspirated and diluted to 1 mL with 0.2 M sodium phosphate buffer (pH 8.0). After that, 2 mL of 0.6 mM DTNB was added. After 10 minutes, the optical density of the yellow-colored complex formed by the reaction of GSH and DTNB (Ellman's reagent) was measured at 405 nm. A standard curve was obtained with standard reduced glutathione. The levels of GSH were expressed as *μ*g of GSH mg^−1^ protein.

### 2.12. Determination of Oxidized Glutathione (GSSG)

The oxidized glutathione level was measured after the derivatization of GSH with 2-vinylpryidine according to the method of Dey and Roy [16]. In brief, with 0.5 mL sample, 2 *μ*L of 2-vinylpryidine was added and incubated for 1 h at 37°C. Then, the mixture was deproteinized with 4% sulfosalicylic acid and centrifuged at 1,000 ×g for 10 min to settle the precipitated proteins. The supernatant was aspirated, and GSSG level was estimated with the reaction of DTNB at 412 nm in spectrophotometer and calculated with standard GSSG curve. The levels of GSSG were expressed as *μ*g of GSSG mg^−1^ protein. All measurements were repeated at least three times.

### 2.13. Nitric Oxide Release Assay

After treatment schedule, 100 *μ*L of Griess reagent (containing 1 part of 1% sulfanilamide in 5% phosphoric acid and 1 part of 0.1% of N-C-1 naphthyl ethylene diaminedihydrochloride) was added to 100 *μ*L of supernatant incubated at room temperature for 10 minutes; readings were taken in a UV spectrophotometer at 550 nm and compared to a sodium nitrite standard curve (values ranging between 0.5 and 25 *μ*M). The levels of NO were expressed as *μ*M mg^−1^ protein [16]. All measurements were done in triplicate.

### 2.14. Intracellular ROS Measurement

ROS measurement was performed using H_2_DCFDA according to the method of Ling et al. [17]. In brief, 96-well plates were seeded with 2 × 10^4^ cells per well and allowed to adhere overnight. In all, 2, 5, and 10 mM safingol added for the indicated period. H_2_O_2_ was used as a positive control. Following the drug treatment, the media were removed and cells were loaded with 5 mM H_2_DCFDA diluted in clear media for 30 mins at 37°C and washed three times using PBS (1X), and then, DCF fluorescence was determined at 485 nm excitation and 520 nm emission using a Hitachi F-7000 fluorescence spectrophotometer. DCF fluorescence was also observed by fluorescence microscopy. DCF-fluorescence intensity of untreated cells was set to 1.00. All measurements were done in triplicate.

### 2.15. Measurement of Mitochondrial Membrane Potential (ΔΨ*m*)

The alteration of mitochondrial membrane potential by spectrofluorometric method was done according to the previously reported method [18]. Briefly, both the control and experimental cells exposed to MMTC at 25 *μ*g mL^−1^ for 18 hr, were washed and suspended in ice-cold PBS. Approximately 1 × 10^6^ cells mL^−1^ number of cells were incubated with 10 mM Rh 123 at 37°C for 30 min and then washed twice with PBS. The cellular fluorescence intensity of Rh 123 was monitored for 2 min using Hitachi F-7000 fluorescence spectrophotometer. The cellular mitochondrial membrane potential was expressed as a percentage of control cells at an excitation wavelength of 485 nm and an emission wavelength of 530 nm. Both excitation and emission slit width were set to 5.0. All measurements were done in triplicate.

### 2.16. DNA Fragmentation Study by Agarose Gel Electrophoresis

DNA gel electrophoresis was performed as described in our previous lab report [19]. In brief, after the treatment schedule, the cells were resuspended in 270 *μ*L precooled lysis buffer (10 mM Tris-Hcl, 10 mM NaCl, and 10 mM EDTA, pH 7.4) with 30 *μ*L SDS (10%). RNase A (final concentration 100 *μ*g mL^−1^) was then added, and incubation was continued at 45°C for 45 min. Subsequently, proteinase K (final concentration 100 *μ*g/mL) was added to the cell lysate, and incubation was continued at 50°C for overnight to complete digestion. DNA was isolated from the lysate using phenol/chloroform/isoamyl alcohol. Then, DNA was precipitated with one volume of 10 M sterile ammonium acetate, and two volumes of absolute ethanol followed by centrifugation at 13000 ×g for 10 min at 4°C. The extracted DNA samples were washed with 70% ethanol and dissolved in 50 *μ*L TE buffer (10 mM Tris-Hcl, 1 mM EDTA, pH 7.6). Gel loading buffer (10 mM EDTA, 0.25% bromophenol blue, and 30% glycerol) was then added to the DNA sample at 1 : 5 ratio and loaded onto a 1.2% agarose gel. The electrophoresis was carried out at 50 V for 90 min in TBE buffer (90 mM Tris-Hcl, 2 mM EDTA, and 90 mM boric acid, pH 8.0). After electrophoresis, DNA was visualized by soaking the gel in TBE buffer containing 1.5 *μ*g mL^−1^ ethidium bromide in UV light, and the picture was captured in Bio-Rad gel documentation system.

### 2.17. Statistical Analysis

All the parameters were repeated at least three times. The data were expressed as mean ± SEM, *n* = 06. Comparisons between the means of control and the treated group were made by one-way ANOVA test (using a statistical package, Origin 6.1, Northampton, MA, USA) with multiple comparison *t* tests, *P* < 0.05 as a limit of significance.

## 3. Result and Discussion

Leukemia is a life threatening hematological malignancy characterized by uncontrolled cell growth. Transition metals have an important role in biomedical application. Research has shown significant progress in the utilization of transition metal complexes as drugs to treat several human diseases like carcinomas, lymphomas, infection control, anti-inflammatory, diabetes, and neurological disorders [4]. In the present, we successfully prepared mononuclear complex [Mn(Phpyk)_2_(SCN)_2_] by chemical reactions. The single crystals of MMTC suitable for X-ray data collection were obtained from the filtrate after a few days. Yield: 90. Anal. Calcd for C_26_O_2_N_4_S_2_H_18_Mn: C, 58.05; H, 3.35; N, 10.42 (%). Found: C, 58.13; H, 3.28; N, 10.37 (%). IR: *v*(SCN) = 2076 cm^−1^, *ν*(C=O) = 1625 cm^−1^, *ν*(skeletal  vibration) = 1579 cm^−1^.

### 3.1. Crystallographic Data Collection and Refinement

**Figure 1:**
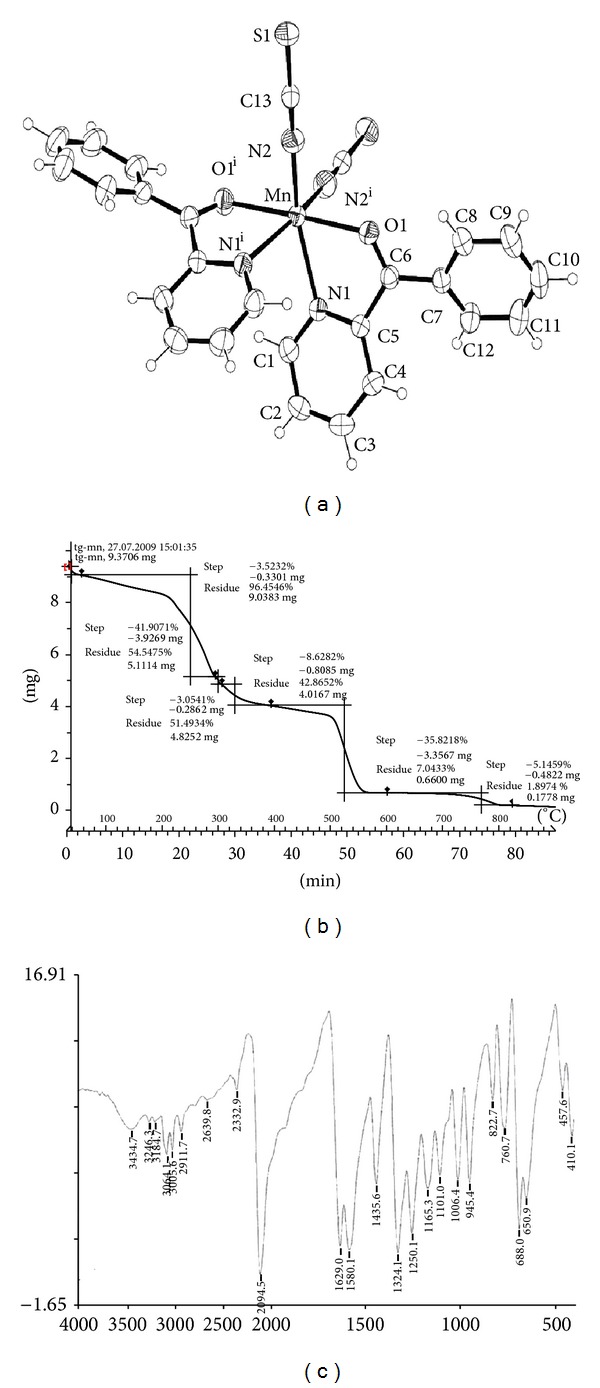
(a) ORTEP drawing (40% probability ellipsoids) of the [Mn(Phpyk)_2_(SCN)_2_] complex (primed atoms at −*x*, *y*, −*z* + 1/2). (b) Thermogravimetric analysis (TGA) of the coordination complex [Mn(Phpyk)_2_(SCN)_2_]. (c) FTIR spectroscopic analysis of the coordination complex [Mn(Phpyk)_2_(SCN)_2_].

Diffraction data for all the structures reported were collected at room temperature on a Bruker Smart Apex diffractometer (Mo-K*α* radiation, *λ* = 0.71073 Å) equipped with a CCD. Cell refinement, indexing, and scaling of the data set were carried out using Bruker Smart Apex packages, and Bruker Saint Package [20]. All the structures were solved by direct methods and subsequent Fourier analyses [21] and refined by the full-matrix least-squares method based on *F* ^2^ with all observed reflections (Figure 1(a)).

### 3.2. Solid State Thermal Studies of the Complex

Solid state thermal analyses have been performed in order to (i) understand the thermal decomposition patterns of the complexes, (ii) to verify the molecular composition of the complexes, and (iii) to synthesize the thermally stable end products. Critical analyses suggest that MMTC yields corresponding metal sulfides as the end products. The thermogram as well as the calculated and experimental weight losses, temperature range of decomposition are reported here (Figure 1(b) and Table 1).

### 3.3. IR Spectral Study

The MMTC exhibits very strong sharp single band in the range of 2094 cm^−1^ which corresponds to the stretching frequency of SCN moiety. The complex exhibits IR stretching frequencies corresponding to the carbonyl group of the hemilabile legend, phpyk at around 1640 cm^−1^ and skeletal vibrations at ~1430 and ~1580 cm^−1^ (Figure 1(c)).

### 3.4. *In Vitro* Cell Proliferation Study

**Figure 2:**
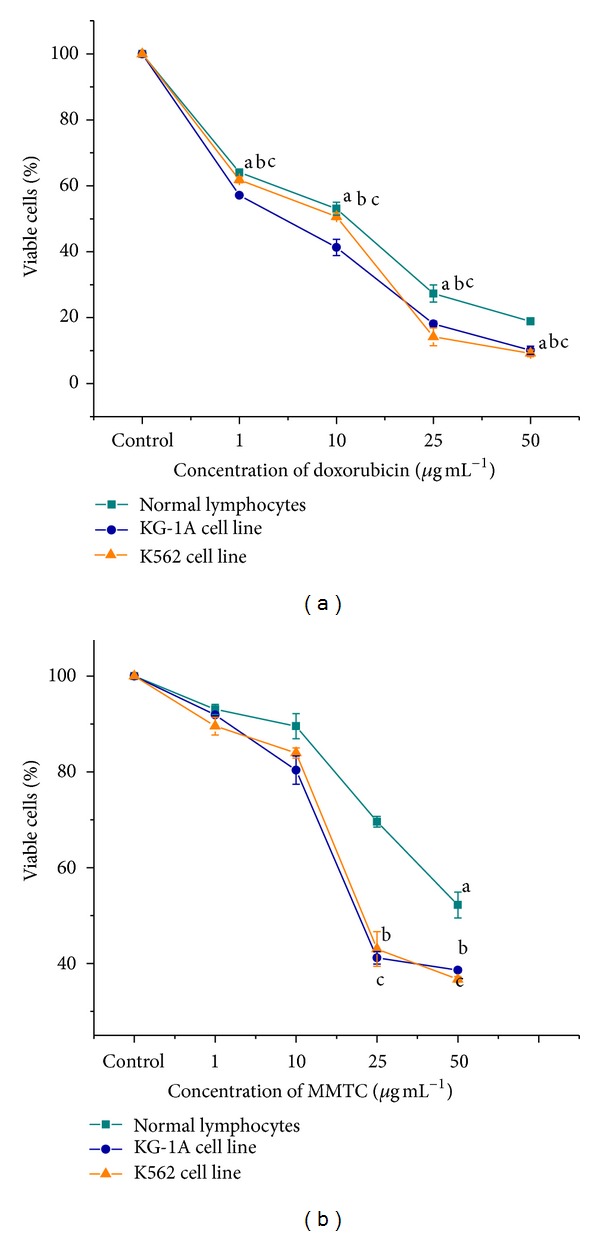
*In vitro* cell proliferation assay of doxorubicin treated (a) and MMTC treated (b) normal lymphocytes, KG-1A and K562 cell lines. Cells were treated with doxorubicin and MMTC for 24 h at 37°C. Cell viability was measured by the MTT method as described in Section 2. Values are expressed as mean ± SEM of three experiments; ∗ and # indicate significant difference (*P* < 0.05) compared to the control group.

Selectivity and good antileukemic efficacy are one of the basic requirements of an anticancer agent. In our study, two types of leukemic cell lines (KG-1A and K562) and normal peripheral blood lymphocytes (PBL) were exposed to DOX and MMTC at the concentrations of 0, 1, 10, 25, and 50 *μ*g mL^−1^ for 24 hours, and cytotoxicity was determined using MTT assay (Figures 2(a) and 2(b)). Results have shown that MMTC up to 25 *μ*g mL^−1^ did not produce any significant (*P* < 0.05) cytotoxic effects in PBL. As the dose increased to 50 *μ*g mL^−1^, cell viability of PBL was decreased significantly by 47.77% due to MMTC exposure. MMTC exposure was significant (*P* < 0.05) and caused a reduction in cell viability in leukemic cell lines by dose dependent fashion. In KG-1A cell line, the viability was significantly decreased by 58.82% and 61.38% at 25 and 50 *μ*g mL^−1^ doses, respectively, whereas in K562 cell line this metal complex was able to reduced cell viability by 56.97% and 63.31% at 25 and 50 *μ*g mL^−1^ doses, respectively. Simultaneously, doxorubicin showed potential toxic effects on normal PBL. It decreased the viability of PBL significantly (*P* < 0.05) by 46.93%, 72.69%, and 81.08% at 10, 25, and 50 *μ*g mL^−1^ doses, respectively. In leukemic cell lines DOX significantly (*P* < 0.05) decreased cell viability from 10 *μ*g mL^−1^ dose.

From this result, it can be stated that though DOX exerted more potent antileukemic potential, side by side, it showed severe toxic effects on PBL. The cytotoxic effects of DOX toward normal cells have already been reported by many researchers [12, 22]. In our study, manganese (II) containing metal complex showed potent antileukemic effects in both cell lines which is supported by many researchers [23, 24]. On the other hand, MMTC did not produce any significant (*P* < 0.05) toxic effects toward healthy cells up to 25 *μ*g mL^−1^ dose. Collectively, these results demonstrate that this dose can be taken as an effective dose for antileukemic therapy using MMTC. So, further experiments were done using this dose only.

### 3.5. Intracellular Uptake by Fluorescence Imaging

**Figure 3:**
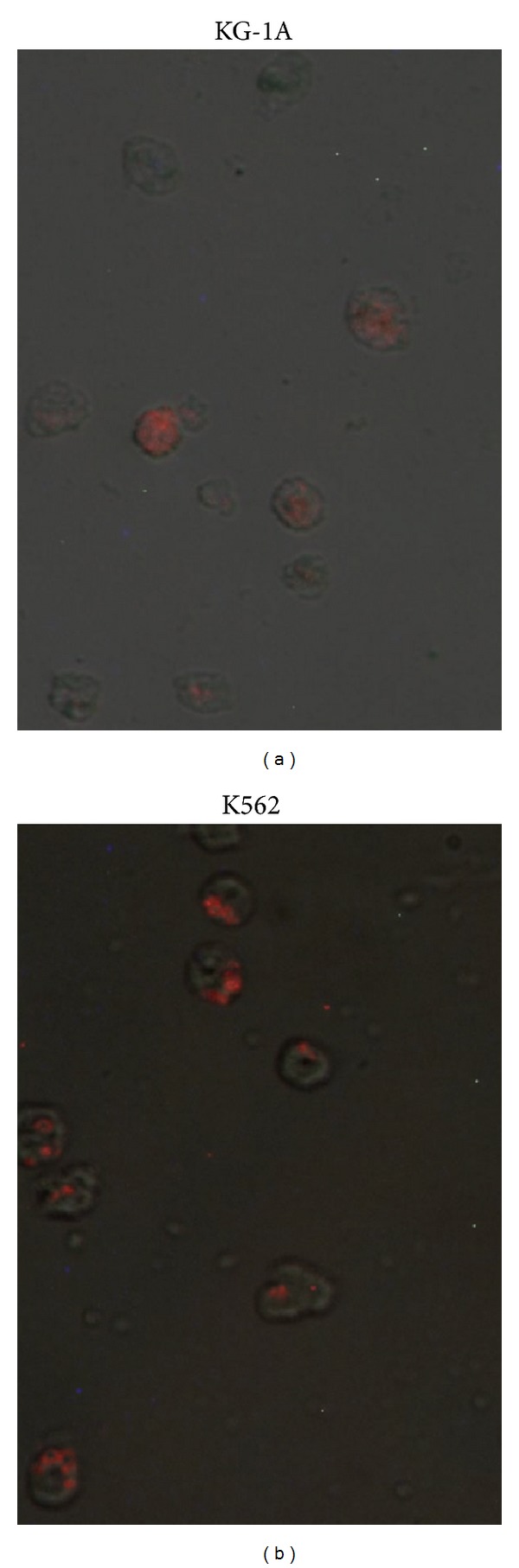
Intracellular uptake of MMTC in KG-1A and K562 cell lines by fluorescence imaging. A required amount of cells was treated with Rhodamine B labeled MMTC for 6 h. Intracellular uptake was examined using fluorescence microscope.

Metal complexes binding to the plasma membrane and intracellular uptake are probably a necessary condition for their exertion of cytotoxicity. We have shown that MMTC was taken up by KG-1A and K562 cell lines in *in vitro* cultures (Figure 3). From the fluorescence micrographs, it was found that fluorescence labeled MMTC was distributed throughout the cytoplasm, indicating the successful uptake of complex in the cells [12]. The internalization processes of MMTC may be due to phagocytosis, pinocytosis, or endocytosis, have all been well studied and seem to strongly depend on particle form, size and cell type used [25]. Effective cellular uptake of any antileukemic drug is necessary for the exertion of potent anti proliferative effects. 

### 3.6. Cellular Redox Status (GSH and GSSG Levels)

**Figure 4:**
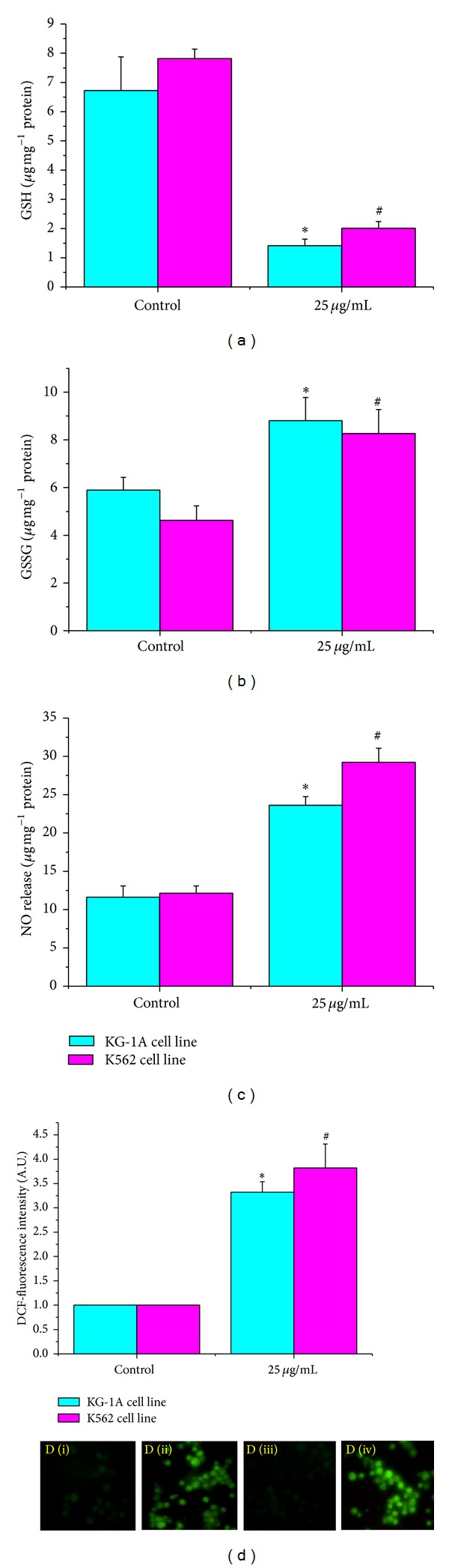
(a) Intracellular reduced glutathione (GSH) levels of MMTC treated KG-1A and K562 cell lines. The levels of GSH were expressed as **μ*g* of GSH mg^−1^ protein. (b) Intracellular oxidized glutathione (GSSG) levels of MMTC treated KG-1A and K562 cell lines. The levels of GSSG were expressed in terms of *μ*g of GSSG mg^−1^ protein. Nitric oxide (NO) release levels of MMTC treated KG-1A and K562 cell lines. (c) The levels of NO were expressed as *μ*mol mg^−1^ protein. (d) Effects of MMTC on ROS induction in KG-1A and K562 cell lines. Data is represented as the percentage of the ROS level in the control group. Values are expressed as mean ± SEM of three experiments; ∗ and # indicate significant difference (*P* < 0.05) compared to the control group. (D (i), D (ii), D (iii), and D (iv)) Qualitative characterization of reactive oxygen species formation by DCFH_2_-DA staining using fluorescence microscopy. Here, D (i): Control KG-1A cells; D (ii): MMTC treated KG-1A cells; D (iii): Control K562 cells; D (iv): MMTC treated K562 cells.

Glutathione is an important antioxidant in the cellular system [26]. Many of the anticancer agents trigger the glutathione system. So, to understand the glutathione level, we have measured both the reduced glutathione (GSH) and the oxidized glutathione (GSSG). Exposure with MMTC decreased the reduced glutathione (GSH) level in both leukemic cell lines. In KG-1A cell line, GSH level was significantly (*P* < 0.05) decreased by 78.97%, and in the case of K562 cell, lines it was decreased by 74.35% at 25 *μ*g mL^−1^ dose (Figure 4(a)). The GSSG level was also elevated significantly (*P* < 0.05) in MMTC treated leukemic cells by 49.46% (in KG-1A cell line), and 78.78% (in K562 cell line) at the same dose (Figure 4(b)).

Glutathione acts as an important intracellular reductant, and it functions as a direct reactive free radical scavenger. In this study, decreased GSH level may be due to increasing level of lipid oxidation products which may be associated with the less availability of NADPH required for the activity of GR to transform GSSG to GSH due to the increased production of free radicals at a rate that exceeds the ability to regenerate GSH in cells [26]. Thus, it can be understood that this MMTC kills leukemic cells by generating oxidative stress and altering cellular redox homeostasis [8].

### 3.7. Nitric Oxide Release Level

In a biological system, the free radical NO is an intra- and inter-cellular messenger [27].  Figure 4(c) showed that MMTC exposure significantly increased NO level in leukemic cells at 25 *μ*g mL^−1^ dose. In KG-1A cell line by MMTC was able to elevate NO level by 103.19%, and 140.67% in K562 cell line. 

NO reacts with superoxide to form a more toxic peroxynitrite (ONOO^−^), which has an important microbicidal and tumoricidal function. In our study, the elevated level of NO in leukemic cells may be due to severe oxidative injury which in turn helped in cell killing ability due to MMTC exposure [28].

### 3.8. Cellular ROS Level

Leukemic cells (KG-1A and K562 cell lines) exposed to MMTC in 24 hours showed increased ROS formation as evidenced by the increased DCF fluorescence intensity. In our study, MMTC exposure significantly (*P* < 0.05) increased ROS generation level by 232% in KG-1A cell line and by 282% in K562 cell line at the effective dose (Figure 4(d) and D (i)–D (iv)).

Reactive oxygen species (ROS) are molecules and ions containing unpaired valence shell electrons, and being free radicals they are highly active and show an important role in cell signaling, leading to oxidative cell damage [29]. In the normal cell, glutathione effectively eliminates ROS and thereby maintain cellular normal metabolic behavior. In our study, increased level of ROS in leukemic cells may be due to the increased conversion of GSH to GSSG [26]. This finding indicates that the MMTC kills the leukemic cells by generating ROS level.

### 3.9. Alterations of Mitochondrial Membrane Potential (MMP)

**Figure 5:**
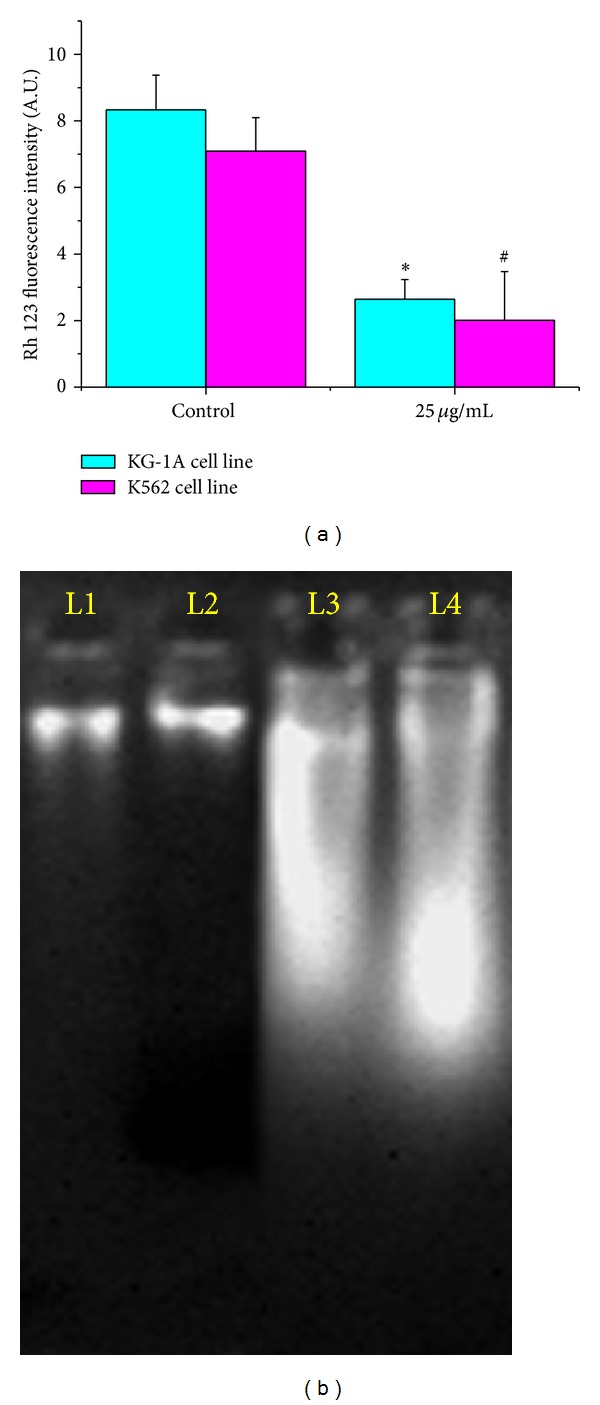
(a) Measurement of mitochondrial membrane potential (MMP) of MMTC treated KG-1A and K562 cell lines. Data is represented as the percentage of the MMP level in the control group. Values are expressed as mean ± SEM of three experiments; ∗ and # indicate significant difference (*P* < 0.05) compared to the control group. (b) DNA fragmentation study by agarose gel electrophoresis. Here, L1: untreated KG-1A cells; L2: untreated K562 cells; L3 and L4: MMTC treated KG-1A and K562 cells, respectively.

One of the possible mechanisms involved in the apoptosis of KG-1A cell line and K562 cell line induced by MMTC was the loss of mitochondrial membrane potential. Mitochondrial membrane potential was determined in terms of Rhodamine 123 fluorescence intensity. In this study, MMTC caused depletion of MMP in both cell lines. In KG-1A cell line, MMP was decreased by 68.27% and in K562 cell line by 71.75% (Figure 5(a)).

Loss of MMP and increased DNA fragmentations are the most common targets for apoptosis induced cell death [30]. Alteration of ΔΨm in MMTC treated leukemic cells may be due to a malfunction in ATP synthesis and maintenance of ATP level that leads to either apoptosis or necrosis [31]. In our study, depleted level of MMP may suggest that MMTC was capable of killing the leukemic cells by induction of apoptosis phenomenon.

### 3.10. DNA Fragmentation

DNA fragmentation study in terms of agarose gel electrophoresis revealed that MMTC was able to interact with the DNA of leukemic cells. Qualitative gel documentation image (Figure 5(b)) suggested that a 25 *μ*g mL^−1^ dose of MMTC for 24 h was capable of fragmenting the DNA strands of KG-1A and K562 cell lines, indicating the involvement of oxidative injury and apoptosis.

The DNA fragmentation is caused by the activation of an endogenous nuclear endonuclease, which selectively and distinctively cleaves the double-stranded nuclear DNA at sites located between nucleosomal units (linker DNA), generating mono- and oligonucleosomal DNA fragments [32]. In our study, elevated level of RNS and ROS may contribute to severe genotoxic effects in leukemic cell lines. 

## 4. Conclusion

In this study, we have demonstrated that MMTC exhibited antileukemic activity in AML and CML cells by dose-dependent manner, whereas it appeared to be less cytotoxic in human normal lymphocytes up to a certain dose. Involvement of oxidative stress mediated by RNS and ROS, alteration of cellular redox status, loss of mitochondrial membrane potential, and genotoxic effects were observed due to MMTC exposure. Based on these results, it can be concluded that oxidative stress is the possible antileukemic mechanism underlying the etiology of cytotoxicity and apoptosis induced by MMTC. However, further studies are necessary to reduce the cytotoxic effects of MMTC in normal cells and to understand the mode of action of such a complex in leukemic cells.

## Figures and Tables

**Table 1:** Thermal analyses data of the coordination complex [Mn(Phpyk)_2_(SCN)_2_].

Complex	Initial temp. of dec. (°C)	Final temp. of dec. (°C)	Calculated weight loss (%)	Experimental weight loss (%)
[Mn(Phpyk)_2_(SCN)_2_]	210	540	83.8	92.8
